# Role of Tet2 in Regulating Adaptive and Innate Immunity

**DOI:** 10.3389/fcell.2021.665897

**Published:** 2021-06-17

**Authors:** Jiaqi Li, Lifang Li, Xiaoxiao Sun, Tuo Deng, Gan Huang, Xia Li, Zhiguo Xie, Zhiguang Zhou

**Affiliations:** ^1^National Clinical Research Center for Metabolic Diseases, Key Laboratory of Diabetes Immunology (Central South University), Ministry of Education, Department of Metabolism and Endocrinology, The Second Xiangya Hospital of Central South University, Changsha, China; ^2^Department of Ultrasound, The Third Xiangya Hospital of Central South University, Changsha, China

**Keywords:** ten-eleven translocation-2, DNA methylation, gene regulation and expression, immune cell differentiation, autoinflammatory and autoimmune diseases

## Abstract

Accumulated evidence indicates that epigenetic modifications play central roles in gene expression regulation and participate in developing many autoimmune and autoinflammatory diseases. Mechanistically, epigenetic modifications act as a bridge between environmental and cellular factors and susceptibility genes. DNA methylation is a critical epigenetic modification that is regulated by ten-eleven translocation (TET) enzymes. Accumulating evidence has revealed that TET family proteins function as gene regulators and antitumor drug targets mainly because of their ability to oxidize 5-methylcytosine (5mC) to 5-hydroxymethylcytosine (5hmC). Recently, the effect of Tet2, an essential TET protein, on the development of autoimmune diseases has been explored. In this review, we summarize the current understanding of Tet2 in immune response regulation, clarify the mechanisms of Tet2 in B and T cell differentiation and function, and discuss the opposing effects of Tet2 on inflammatory gene expression in the immune system to provide new potential therapeutic targets for related diseases.

## Introduction

DNA methylation is an epigenetic modification that can regulate gene expression without affecting the DNA sequence (Xie et al., 2018). DNA methylation is influenced by both environmental and genetic factors and has gradually been proposed to affect the regulation of key genes involved in autoimmunity and its variation in related diseases (Husquin et al., 2018). DNA methylation occurs widely in archaea, bacteria and eukaryotes but not in yeast. In mammals, DNA methylation sites are located mainly on cytosine-guanine dinucleotide (CpG) sites, and a low level of methylation on non-CpG sites is found in embryonic stem cells (ESCs). In the human genome, the percentage of methylated CpG sites is 70∼80%, while unmethylated CpG dinucleotides are enriched mainly in gene promoter regions and usually exist in clusters called CpG islands (CGIs) (Jaenisch and Bird, 2003; Husquin et al., 2018). Evidence has indicated that DNA methyltransferases (DNMTs) are necessary in mammals to establish and maintain DNA methylation. DNMTs bind to DNA and use S-adenosylmethionine as a methyl donor to catalyze the formation of 5-methylcytosine (5mC). Additionally, the level of DNA methylation is controlled by the balance between DNA methylation and demethylation. Two mechanisms are involved in subsequent DNA demethylation: replication-independent active DNA demethylation and replication-dependent passive DNA demethylation. Recently, knowledge about ten-eleven translocation (TET) demethylase family mediated active DNA demethylation has improved (Kohli and Zhang, 2013).

**FIGURE 1:**
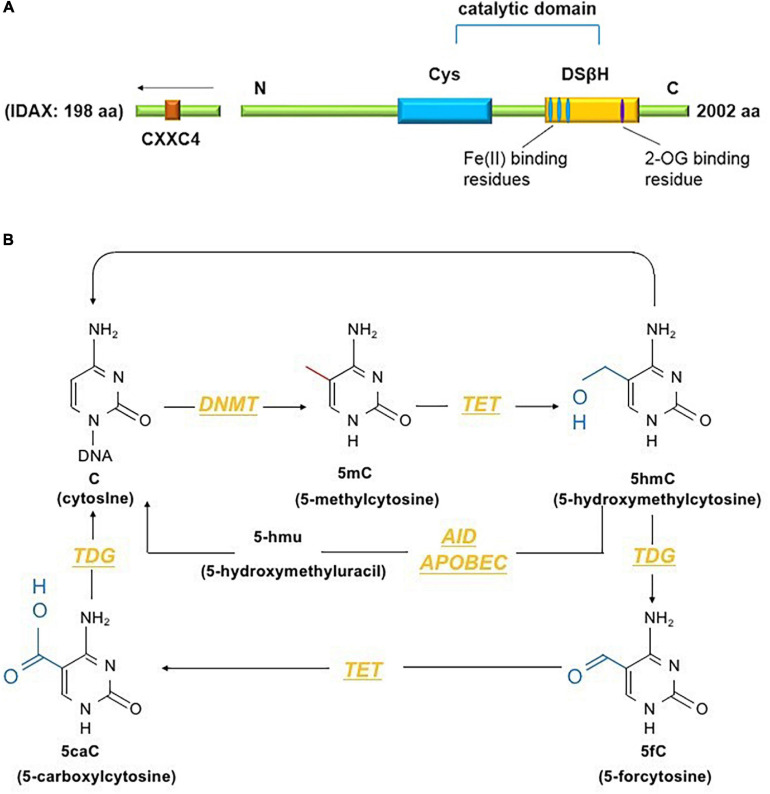
**(A)** The conserved structural domains of Tet2 proteins include a cysteine (Cys)-rich domain and a double-stranded β-helix fold (DSβH), which is characteristic of Fe(II) and 2-oxoglutarate binding and is required for the catalytic activity of Tet2 (Feng et al., 2019). Additionally, the CXXC domain of Tet2 is encoded by the independent IDAX gene, which is also called CXXC4 (Li and Xu, 2019). **(B)** DNA methyltransferases (DNMTs) methylate unmodified cytosine (C) residues at the 5 position to form 5mC. The Tet proteins then convert 5mC to 5hmC, 5fC, and 5caC (considered active modifications), and 5hmC can be deaminated to form 5hmU via the AID- and APOBEC-mediated pathways (Solary et al., 2014).

The TET family comprises dioxygenases containing a catalytic domain that consists of a cysteine (Cys)-rich domain and a double-stranded β-helix fold (DSβH) at the C-terminus. The DSβH domain contains an αKG (also called 2-oxoglutarate) binding site and three iron (Fe2^+^) binding sites (Figure 1A). TET proteins contribute to DNA demethylation by iteratively catalyzing the conversion of 5mC to 5-hydroxymethylcytosine (5hmC), 5-formylcytosine (5fC) and 5-carboxylcytosine (5caC). Next, 5mC is converted back to unmodified cytosine by base excision repair (BER) via the DNA glycosidase thymine-DNA glycosylase TDG (Ito et al., 2011). The TET family has three members – Tet1, Tet2 and Tet3. Among them, Tet2, the second TET family member located on chromosome 4q24, serves as an important regulator in the immune response (Cong et al., 2021). Tet2 was first identified in myeloid malignancies (Delhommeau et al., 2009) and is considered to function as a DNA-modifying enzyme to oxidize 5mC to 5hmC (Figure 1B).

Immunofluorescence analyses showed that Tet2 is exclusively expressed in naïve ESCs and controls the methylation of specific genomic regions in these cells (Pantier et al., 2019). Additionally, cells are reprogrammed from a primed to a naïve pluripotency state through ectopic expression of Tet2 (Fidalgo et al., 2016), and Tet2 functions in promoting enhancer demethylation by interacting with CCAAT/enhancer-binding protein alpha (C/EBPα), Krüppel-like factor 4 (KLF4) and transcription factor cellular promoter 2-like 1 (TFCP2L1) in different stages of induced pluripotent stem cell reprogramming (Sardina et al., 2018). Furthermore, the Tet2 gene coding region, splice sites, and other evolutionarily conserved regions are destroyed by various mechanisms, such as the accumulation of nonsense, missense, or frameshift mutations, all of which may cause partial or total loss of Tet2 function (Patnaik et al., 2016).

Although TET proteins have overlapping effects on DNA methylation, their contributions to generating 5hmC in different cell types differ. For example, compared with other TET proteins, Tet2 accounts for the greatest proportion of 5hmC generation in mouse ESCs (Huang et al., 2014). Chromatin-related proteins contain a CXXC structural domain that plays an important role in distinguishing between methylated and unmethylated DNA. Although Tet2 does not contain a CXXC structural domain like Tet1 and Tet3, this domain is encoded by an independent IDAX gene, which is also called CXXC4 and participates in the interaction between Tet2 and DNA (Tahiliani et al., 2009). Through activation of caspase-related pathways, IDAX functions in decreasing the Tet2 protein level (Ko et al., 2013). Patients with hematological malignancies who also have Tet2 mutations or loss of function are more likely to develop autoimmune and autoinflammatory diseases than those without Tet2 mutations, and these abnormalities can even cause various tumors (Pan et al., 2015). Over the last decade, numerous studies have illuminated the biological functions of Tet2 in malignancies (Ko et al., 2015; Pan et al., 2015; Bowman and Levine, 2017). In this review, we focus on the role of Tet2 in immune cell development and function. We also highlight our current understanding of Tet2 in regulating the adaptive and innate immune systems and discuss basic and clinical approaches to derive benefit from Tet2 while avoiding its liabilities.

## Role of Tet2 in B-Cell Development and Function

The process of B-cell development is identified as a complex program that integrates internal and environmental signals (Kurosaki et al., 2010). Among this process, B cells undergo a series of stages; they start from hematopoietic stem cells in the bone marrow and differentiate into multipotent progenitors and common lymphoid progenitors. The B-cell lineage is then committed, including pro-B cells, pre-B cells, mature naïve B cells and mature B cells (Slifka et al., 1998; Lio and Rao, 2019). When mature naïve B cells migrate to peripheral lymphoid organs and are exposed to an antigen, the corresponding antigen-specific B cells are activated under the stimulation of “dual signals” comprising B-cell receptor (BCR) antigen recognition and Th cell assistance (Cong et al., 2021). With the help of cytokines produced by Th cells and T follicular helper (Tfh) cells, activated B cells proliferate to form germinal centers (GCs). By undergoing somatic hypermutation, immunoglobulin (Ig) affinity maturation and Ig class switching (also called isotype switching), B cells differentiate into plasma cells or memory B cells, which provide the full range of humoral immune functions (Bossen et al., 2012; Alt et al., 2013).

Tet2 is expressed and plays a pivotal role in the progression of B-cell differentiation. Conditional Tet2/Tet3 knockout largely prevents lineage-specific programmed demethylation and then causes the destruction of B-cell differentiation and function (Orlanski et al., 2016). The loss of Tet2 and Tet3 in mice at an early B stage results in the prevention of pro-pre B cell transition by modulating the modification status of DNA (Lio et al., 2016). Additionally, during the progression of B-cell maturation and activation, Tet2 also plays an important role (Schoeler et al., 2019). These results suggest that it is essential to clarify the definite mechanism of Tet2 in a series of B-cell developmental stages.

### Tet2 Is Involved in the Pro-B to Pre-B Transition

The loss of demethylation might affect early B-cell development and function (Lio et al., 2016; Orlanski et al., 2016). Tet2/Tet3 double knockouts (DKOs) are associated with a partial blockade of the transition from pro-B cells to pre-B cells. Compared with the controls, the ratio of pro-B cells to pre-B cells in the bone marrow of Tet2/Tet3 DKO mice showed a threefold change (Orlanski et al., 2016). Flow cytometric analyses showed that the number and percentage of a series of B cells were also significantly changed. A study conducted by Orlanski et al. revealed that the absence of demethylation caused by Tet2/Tet3 DKO leads to an accumulation of primordial cells, which affects the process of B-cell maturation and is accompanied by a striking reduction in the percentages and numbers of recirculating (mature) B cells. These trends were also found in the spleen, and Tet2/Tet3 DKO is associated with a lack of mature B-cell subsets, such as marginal zone (MZ) B and B1 cells, accompanied by many abnormal pro-B cells (Orlanski et al., 2016). Interestingly, a similar study carried out by Lio et al. showed a different result for the changes of pro-B cells, suggesting that although the percentage of pro-B cells increased in the bone marrow of Tet2/3 DKO, the total number of pro-B cells was unaltered due to the overall decrease in total B-lineage cells. Therefore, the changes in pro-B cells in Tet2/Tet3 DKO remain to be fully elucidated. More specifically, it was pointed out that in Tet2/3 DKO bone marrow, the degree of reduction of B220^+^ CD19^+^ cells changed with time. At 7–8 weeks of age, the percentage of B220^+^ CD19^+^ cells reached less than 50% of wild-type (WT) bone marrow, and it was reduced to less than 10% at 11–12 weeks of age. In addition, the percentages of pre-B cells and immature B cells in the Tet2/3 DKO bone marrow were substantially reduced (7–20% of that in WT bone marrow) at 11–12 weeks (Lio et al., 2016).

At the early stages of B cell development, the consensus binding motifs of key B-lineage transcription factors are enriched in enhancers via the induction of focal DNA hypomethylation at enhancers by the combination of Tet2 and Tet3, and the gene expression program related to maturation transition is enhanced (Manoharan et al., 2015; Lio et al., 2016). A susception has been demonstrated that Tet2/Tet3 DKO-induced gene expression of developmental factors occurs in the early pro-B stage (Choukrallah and Matthias, 2014). A functional relationship has been found between key transcription factors and TET proteins during B cell development. There are two key transcription factors (E2A and PU.1) at Eκ enhancers in pro-B cells that play a positive role in maintaining the demethylated status of these enhancers (Ghisletti et al., 2010). E2A and PU.1 coimmunoprecipitated with Tet2 via the recruitment of Tet2 to Eκ enhancers. PU.1 can serve as a “pioneer” transcription factor located upstream of TET proteins and then functions by recruiting TET and then interacting with it to increase the accessibility of bound enhancers. Interestingly, the expression of another transcription factor, IRF4, is also regulated by TET proteins; without the catalytic activity of Tet2 and Tet3, IRF4 cannot restore Ig kappa (Igκ) germline transcription or the demethylation status of Igκ enhancers (Lio et al., 2016). Moreover, the hypothesis of an interaction of Tet2 and early B-cell factor 1 [EBFI, a key transcription factor in the regulatory network at the pre-pro-B cell stage (Reynaud et al., 2008)] in SW1353 cells has been identified. Coimmunoprecipitation and western blotting demonstrated that there was an endogenous interaction between Tet2 and EBF1. As a potential candidate interactor of Tet2, EBF1 cooperates with Tet2 to contribute to DNA demethylation in B cells via a sequence-specific mechanism. In addition, during the progression of B-cell development dysregulation and B-cell malignancy pathogenesis, 5hmC alteration caused by Tet2 inactivation could promote this interaction (Guilhamon et al., 2013). Collectively, these findings suggest that the loss of function of Tet2 and Tet3 might impair the transition from pro-B cells to pre-B cells in B-cell development (Lio et al., 2016).

The main events in early B-cell development in the bone marrow are the expression of the BCR and development of B-cell immune tolerance (Winkler and Martensson, 2018). Gene groups encoding BCRs exist in the form of gene segments at the germline stage. To produce large amounts of BCRs that can recognize specific antigens, rearrangement of the genes encoding the Ig heavy and light chains is needed. Gene rearrangement of Igκ light chains *in vivo* occurs at the pre-B cell stage (Shinnakasu et al., 2016). Evidence has shown that both Tet2 and Tet3 contribute to Igκ rearrangement by demethylating and modulating the activity of Igκ enhancers and altering κ chain expression in pre-B cells, thus playing a positive role in the production of B cells with functional BCRs (Lio et al., 2016).

### Tet2 Function in B-Cell Maturation and Activation

Both Tet2 and Tet3 can participate in transcriptional reprogramming, function as regulators of DNA methylation to maintain GC and determine the terminally differentiated fate of B cells. Under the guidance of Tet2 and Tet3, GC B cells differentiate into plasma cells that secrete substantial amounts of high-affinity antibodies, including lgG and lgλ (Hobeika et al., 2006; Wille et al., 2017; Schoeler et al., 2019). Deficiency of Tet2 and Tet3 leads to GC hyperplasia, suppresses plasma cells and further promotes the formation of B-cell lymphoma (Schoeler et al., 2019). Although Tet2/Tet3 DKO prevents plasmacytic differentiation, it does not impair or depend on cell proliferation (Caron et al., 2015).

**FIGURE 2:**
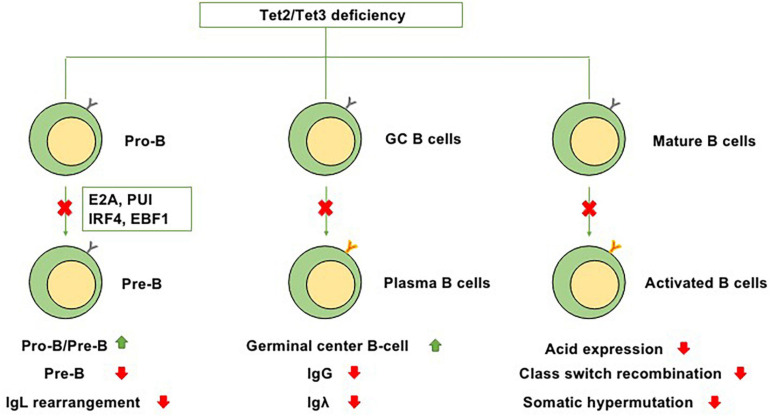
Functions of Tet2/Tet3 in B-cell differentiation. Because of Tet2/Tet3 deficiency, (1) B-cell development at the pro-B to pre-B transition is impaired. (2) It causes germinal center hyperplasia, impairs plasma cell differentiation, suppresses IgG and Igλ expression, and promotes B-cell lymphomagenesis. (3) During the transition from mature B cells to activated B cells, Tet2/Tet3 deficiency decreases the expression level of Acida and then downregulates the expression of AID, thus impairing Ig class switching and somatic hypermutation.

At the transition from mature B cells to activated B cells, enzyme activation-induced cytidine deaminase (AID), which is encoded by Acida, is required for somatic hypermutation and Ig class switching (Lio and Rao, 2019). In GC B cells, AID is present on both Ig and non-Ig loci, obviously affecting DNA hypomethylation (Dominguez et al., 2015). A cooperative relationship exists between TET enzymes and AID-mediated cytosine demethylation, and statistical analysis has indicated that a marked number of overlapping CpGs are regulated by both Acida and Tet2 (Pfaffeneder et al., 2014). In murine B cells, Tet2 and Tet3 can increase the expression level of Acida and then upregulate the expression of AID and conversion of 5hmC, suggesting that the cooperation between Tet2 and Tet3 plays a role in regulating Ig class switching (Lio and Rao, 2019). However, in the setting of Tet2 single knockout, the expression of Acida and levels of 5mC and 5hmC are only slightly perturbed, suggesting that in the setting of Tet2 single knockout, the suppression of Acida-mediated demethylation of these residues, not the effects on the expression of Acida itself, leads to the significant failure to demethylate Acida-regulated CpGs (Rosikiewicz et al., 2020). Collectively, these studies indicate that TET proteins, particularly Tet2, play regulatory roles in B-cell development at each stage (Figure 2).

## Tet2 Regulates T Cell Development and Function

**FIGURE 3:**
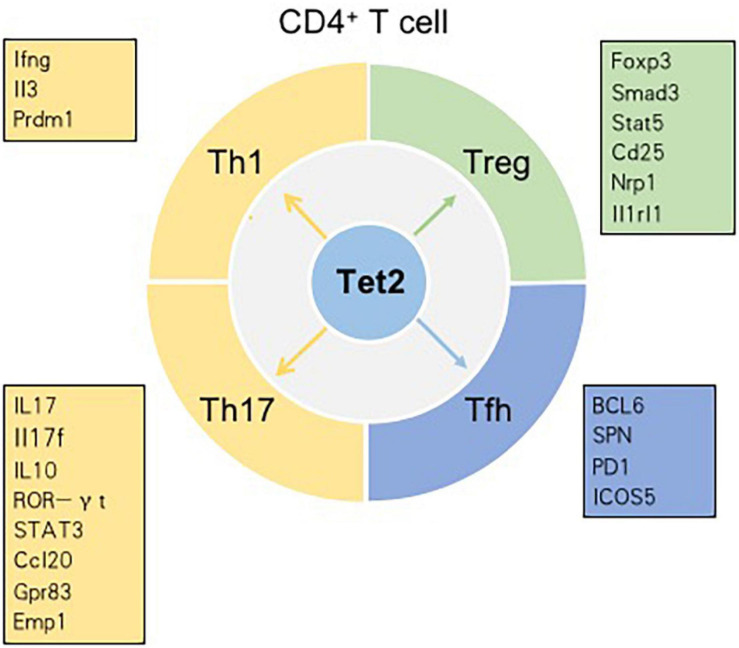
Tet2 modulates the differentiation of CD4^+^ T cell subsets in a context-dependent manner. Genes in the boxes are involved in the differentiation of CD4^+^ T cell subsets regulated by Tet2.

T cells, originating from hematopoietic stem cells, proceed through a series of developmental stages from lymphoid progenitor cells to pro-T cells, pre-T cells, immature T cells and mature T cells in the thymic microenvironment. The functions of mature T cells not only mediate the adaptive cellular immune response but also play an important auxiliary role in the thymus-dependent antigen-induced humoral immune response. Therefore, T cells are critical for the adaptive immune response. In the thymus, the most essential events for T cell development are the acquisition of diverse T cell antigen receptors (TCRs), autogenous MHC restriction (positive selection), and the formation of immune self-tolerance (negative selection). According to their different functions, T cells can be normally divided into three categories: helper T (Th) cells, cytotoxic T cells (CTLs) and regulatory T cells (Tregs) (Carpenter and Bosselut, 2010; Yui and Rothenberg, 2014). Tet2 has been demonstrated to play an indispensable role in regulating T cell development and function (Figure 3).

### Effect of Tet2 on Th Cell Differentiation

Generally, Th cells expressing CD4 are called CD4^+^ T cells. Regulated by the characteristic antigens and cytokine signaling acting on lineage-specific transcription factors, Th0 cells differentiate into a series of Th cell lineages (Kanno et al., 2012). Each Th cell subset expresses lineage-specific genes that are strongly related to the levels of 5mC and 5hmC. Some cytokine genes, such as interferon gamma (Ifng), interleukin-4 (Il4), and Il17, have been considered the defining lineage markers for Th1, Th2, and Th17 cells, respectively, and all are closely related to 5hmC (Ichiyama et al., 2015). In naïve CD4^+^ T cells, 5mC is usually located in transcriptional regulatory regions (particularly promoter regions) at the cytokine locus. These regions always overlap with conserved non-coding sequences (CNSs), and these cytokine genes remain silenced. However, 5hmC in promoter regions has a strong positive correlation with gene expression. Therefore, during the differentiation of naïve CD4^+^ T cells into Th cells, TET proteins play a role by oxidizing 5mC in these regions to 5hmC in a lineage-specific manner (Pastor et al., 2013).

CNS6 is an enhancer at the Ifng locus and is highly hydroxymethylated specifically in Th1 cells (Pastor et al., 2013). Tet2 is recruited to CNS6 of Ifng in Th1 cells in a manner dependent on the key lineage-specific transcription factor T-bet. Evidence indicates that Tet2 and T-bet may coordinately regulate Ifng expression. In Th1 cells, genetic deletion of either T-bet or Tet2 reduces the recruitment of the other gene; hence, the expression of the Ifng gene decreases (Ichiyama et al., 2015). CNS2, a known enhancer in the Il17 and Il17f genes, is also hydroxymethylated in Th17 cells and plays an indispensable role in IL-17 lineage-specific expression. Tet2 deficiency results in a decrease in 5hmC in CNS2 and enhancer regions of Il17 and Il17f. Previously, retinoic acid-related orphan receptor (ROR)γt (and RORa) were found to bind CNS2 and then initiate chromatin remodeling at the Il17-17f locus (Wang et al., 2012). In Th17 cells, Tet2 induces DNA demethylation at Il17-17f sites in a RORγt-dependent manner. In addition to being recruited to CNS2 of Il17 and Il17f, Tet2 is also recruited to CNS2 at the Il10 locus in a signal transducer and activator of transcription 3 (STAT3)-dependent manner. Additionally, Tet2 deficiency impacts Th17 cells more strongly than Th1 cells. In the setting of Tet2 deficiency, the depletion of 5hmC around transcription start sites (TSSs) and CpG islands (CpGIs), as well as the decrease in the 5hmC tag density, is much higher in Th17 cells than in Th1 cells. DNA microarray analysis in Tet2^+/+^ and Tet2^–/–^ Th1 cells showed that, among 229 Th1 cell-specific genes, the expression of only Ifng, Il3, and Prdm1 is regulated by Tet2. Similarly, among the 197 Th17 cell-specific genes, only six genes – Ccl20, Il10, Gpr83, Emp1, Rbl2 and Il17 – are regulated by Tet2 (Ichiyama et al., 2015). However, compared with that of Th1 or Th17 cells, the defining phenotype of Th2 cells is not directly related to the genes whose expression is regulated by Tet2. IL-4/13A is a pivotal cytokine in the differentiation and functions of Th2 cells. A zebrafish model proved that Tet1 and Tet3 play significant roles in promoting IL-4/13A induction in response to stimulation with exogenous soluble antigen and then regulate the expression of genes related to Th2 cells (Yang et al., 2016).

In addition to the roles of Th1, Th17, and Th2 cells, the role of Tfh cells cannot be ignored. As a subtype of effector CD4^+^ Th cells, Tfh cells are located in the GC and highly express surface receptors such as CXCR5, B cell lymphoma-6 (BCL-6), PD1 and inducible costimulatory receptor (ICOS) (Crotty, 2014). Tfh cells play an indispensable role in B-cell differentiation and survival and the process of antibody affinity maturation (He et al., 2018). A study pointed out that the key transcription factor BCL-6 is linked to a decrease in 5hmC. BCL-6 promoted Tfh cell differentiation by antagonizing the IL-7R (CD127)/signal transducer and activator of transcription (STAT) 5 axis. Thus, Tfh cell differentiation is regulated by DNA methylation (Liu et al., 2016). High salt (sodium chloride, NaCl) concentrations could promote Tet2-mediated hypomethylation in CD4^+^ T cells and induce the differentiation of Tfh cells by regulating genes related to T cell differentiation, such as SPN (CD43). *In vitro*, Tet2 silencing was found to significantly suppress NaCl-induced Tfh cell polarization (Wu et al., 2016). Angioimmunoblastic T cell lymphoma (AITL), originating from the malignant transformation of Tfh cells, is an aggressive peripheral T cell lymphoma. The combination of Tet2 loss-of-function mutations and a highly recurring mutation of the RHOA small GTPase (p. Gly17 Val) leads to the development of AITL (Cortes et al., 2018). Collectively, most of the mentioned findings demonstrate that Tet2 is crucial in Th differentiation by inducing DNA demethylation and controlling signature cytokine gene expression in T cells.

### Role of Tet2 in Promoting CD8^+^ CTL Differentiation

Cytotoxic T lymphocytes (CTLs) are also called CD8^+^ CTLs because they express CD8, while γδ T cells and natural killer T (NKT) cells, which have the same cytotoxic effect as CTLs, do not belong to the family of CTLs (Sanin and Pearce, 2016). A study performed by the Carty group aimed to explore the function of Tet2 in CD8^+^ T cell fate decisions (Carty et al., 2018). They infected Tet2 conditional knockout (Tet2cKO) and control mice with lymphocytic choriomeningitis virus (LCMV)-Armstrong and then tested the potential of Tet2cKO CD8^+^ T cells to respond to antigenic stimulation. Interestingly, Tet2cKO CD8^+^ T cells exhibited enhanced production of cytokines, such as IFNγ and CD107a (Carty et al., 2018), in contrast to the expectation that cells with Tet2 deficiency would produce fewer cytokines (Cui and Kaech, 2010). Collectively, these data suggest that Tet2 loss plays a vital role in directly influencing CD8^+^ T cell fate in the setting of acute LCMV infection. After acute viral infection in mice with selective Tet2 loss in CD8^+^ T cells, a memory phenotype dependent on cell-intrinsic mechanisms rather than the disruption of antigen-driven cell expansion or effector function is preferentially adopted. Additionally, these cells demonstrate superior pathogen control following secondary recall (Cui and Kaech, 2010). The mechanism by which Tet2 promotes a CD8^+^ T cell memory transcriptional program acts by controlling the expression of key transcription factors such as Eomes and T-bet. Methylation analysis of LCMV-specific CD8^+^ T cells indicated that, compared with WT CD8^+^ T cells, CD8^+^ T cells with Tet2 loss exhibited a small number of differentially methylated regions (DMRs). These DMRs are not located at each locus encoding different effector or memory markers, such as CD27, CD62L, and CXCR3; instead, several hypermethylated regions are considered transcriptional drivers of effector/memory CD8^+^ T cell fate (Carty et al., 2018). Collectively, these data show that Tet2 is a key regulator in the determination of CD8^+^ T cell fate.

### Tet2 Functions in Regulating Foxp3^+^ Treg Cells

Treg cells are a type of T cell characterized by a CD4^+^CD25^+^Foxp3^+^ expression profile. Forkhead box p3 (Foxp3) is the master regulatory transcription factor that shapes the fate of Treg cells, and its deficiency leads to an absence of Treg cells and induction of autoimmunity (Josefowicz et al., 2012). During Treg cell differentiation and function, 3 major cis elements [conserved non-coding DNA sequences, CNSs (CNS1, CNS2 and CNS3)] at the Foxp3 locus encode information that determines the composition, size and maintenance of the Treg cell population (Zheng et al., 2010). The Foxp3 locus contains conserved CpG-rich regions called Treg-specific demethylated regions (TSDRs), and CNS2 was the original TSDR found in this region (Kanno et al., 2012). CNS2 is demethylated in Treg cells; however, in naïve T cells, CNS2 is fully methylated in differentiated T cell subsets. The role of CNS2 is helpful for Foxp3 expression in the progeny of dividing Treg cells, while it has a limited role in Foxp3 induction. Correspondingly, CNS3 can promote the development of T cells in the thymus and periphery. CNS1 contains a transforming growth factor-β (TGF-β)-NFAT response element, which does not obviously affect the differentiation and development of T cells in the thymus but plays a crucial role in promoting the development of peripheral T cells (Zheng et al., 2010).

Ten-eleven translocation proteins play an essential role in regulating the immune homeostasis of Treg cells. During Treg cell development in the thymus, 5mC loss in TSDRs (CNS1 and CNS2) is mediated by TET proteins. Tet1 and Tet2 cooperate to play a positive role in establishing a Treg cell-specific hypomethylation pattern and stabilizing Foxp3 expression by catalyzing the conversion of 5mC to 5hmC in Foxp3. Mice with Tet1 and Tet2 deficiency exhibit CNS2 hypermethylation, impaired Treg cell differentiation and function, and a subsequently increased risk of developing autoimmune diseases. Interestingly, the function of hydrogen sulfide (H_2_S) in Foxp3^+^ Treg cells has been identified. H_2_S deficiency results in the downregulation of Tet1 and Tet2, and the expression of Foxp3 is decreased in Treg cells, as demonstrated by qPCR and Western blot analysis. In CD4^+^ T cells, treatment with the H_2_S donor NaSH rescued the expression of Tet1 and Tet2, whereas treatment with the H_2_S inhibitors hydroxylamine (HA) or DL-propargylglycine (PAG) led to decreased expression of Tet1 and Tet2. Mechanistically, H_2_S sulfhydrates nuclear transcription factor Y subunit beta (NFYB) to facilitate its binding to the Tet1 and Tet2 promoters; Tet1 and Tet2 are then recruited to bind to Foxp3 by TGF-β-activated Smad3 and IL-2-activated Stat5 (Li et al., 2014; Yang et al., 2015), thus maintaining Foxp3 CNS2 demethylation and playing an important role in Treg cell-associated immune homeostasis (Yang et al., 2015). Concomitantly, compromised stability of Foxp3 expression was observed in Treg cells from mice with combined disruption of Tet2 and Tet3 expression. The same result was obtained in CNS2-deficient Treg cells, and CNS1, CNS2 and other TSDRs exhibited DNA hypermethylation (Yue et al., 2016); thus, Treg cell lineage specification and endowment of a stable Treg identity were impaired. Additionally, Treg cells from mice with Treg cell-specific deletion of Tet2 and Tet3 (Tet2/3^*fl*^/^*fl*^Foxp3^*Cre*^) display dysregulated expression of many Treg cell signature genes, including Cd25, neuropilin-1 (Nrp1), and IL-1 receptor-like 1 (Il1rl1), resulting in the loss of their suppressive function (Sawant and Vignali, 2014; Sekiya et al., 2015). After the loss of Foxp3 expression, Treg cells are called “ex-Treg” cells (Zhou et al., 2009). These ex-Treg cells and conventional CD4^+^ and CD8^+^ T cells continue to acquire aberrant effector functions. Notably, the aberrant properties of Tet2/3^*fl*^/^*fl*^Foxp3^*Cre*^ Treg cells are dominant and cannot be prevented or rescued by WT Treg cells, eventually causing a fatal inflammatory/lymphoproliferative disease (Yue et al., 2019). Vitamin C (VC), as a small-molecule activator, not only increases the stability of Foxp3 expression in TGF-β-induced Treg cells but also promotes Tet2 enzyme folding and/or recycling of the cofactor Fe2^+^ by interacting with the C-terminal catalytic domain of Tet2 (Tahiliani et al., 2009), a method proven to be effective for Treg cell-mediated adoptive immunotherapy (Someya et al., 2017). Therefore, Tet2 plays key roles in maintaining TSDR demethylation and Treg cell stability/function.

## Tet2 Functions in Myeloid Cell Differentiation

Bone marrow myeloid cells comprise dendritic cells (DCs), monocytes, macrophages (MACs) and microglia (Stienstra et al., 2017). Because of their plasticity, myeloid cells have various functions, including functions in innate immunity as macrophage (MAC, MΦ) effectors and bone remodeling as osteoclasts (OCs). Some studies have indicated that Tet2 may play a key role in shaping myeloid cell plasticity because of its high expression in these cells and its frequent mutation in myeloid leukemias (Ponciano-Gomez et al., 2017).

Transcriptomic and epigenomic analyses have suggested that macrophages and osteoclasts, terminally differentiated myeloid cells, undergo similar 5hmC and 5mC changes but exhibit differential macrophage- and osteoclast-specific gene expression mediated by the ratio of Tet2 to TDG (Tet2/TDG). Downregulation of Tet2 and TDG impairs not only the DNA methylation process but also the recruitment of various histone-modifying enzymes. Nuclear factor κB (NF-κB) ligand (RANKL)-dependent histone modification is associated with differential demethylated gene patterns, and these genes are sensitive to further epigenetic modifications in macrophages and osteoclasts (Garcia-Gomez et al., 2017). The H3K4 methyltransferase SETD1A binds to these genes in a Tet2-dependent manner, and both TET protein and O-GlcNAc transferase (OGT) activity can promote the binding of SETD1A to chromatin (Deplus et al., 2013). Additionally, 5hmC, as a stable epigenetic mark, can directly stabilize the final myeloid identity and function (Bachman et al., 2014). The abovementioned observations indicate that Tet2 and TDG are crucial contributors in determining the final phenotype of myeloid cells (Garcia-Gomez et al., 2017).

### Tet2 Regulates Inflammatory Gene Expression in Myeloid Cells

Tet2 was identified as a repressor of inflammatory gene expression in myeloid cells (Ko et al., 2010). In response to stimulation, both macrophages and DCs with Tet2 deficiency exhibit increased IL-6 expression (Cull et al., 2017). In mice, Tet2^–/–^ macrophages produce larger amounts of inflammatory factors, such as IL-1β, IL-6, and arginase 1, than WT macrophages. Tet2 is strongly induced during the progression of inflammatory immune activation and significantly inhibits the secretion of the inflammatory cytokine IL-6 at the later stage of inflammation (Zhang et al., 2015). By recruiting histone deacetylases (HDACs), TET proteins are regarded as mediators of transcriptional repression in immune cells (Zhang et al., 2015). Tet2 is related to Iκbζ and binds to the enhancer of the IL-6 gene; Tet2 then recruits histone deacetylase 2 (HDAC2) and represses the expression of IL-6. Because this repression has no direct relevance to the catalytic activity of Tet2, Tet2 mediates the formation of a repressive complex based on a structural scaffold. Additionally, systemic Tet2 loss could lead to impaired intestinal barrier function. Under this condition, bacteria are translocated from the impaired intestinal lumen to internal organs, accompanied by IL-6 production and inflammation (Meisel et al., 2018). Therefore, mice with Tet2 deficiency may show different basic levels of inflammation consistent with the specific capabilities of the microbiota. This feature may explain the variable phenotypes of different strains of Tet2-deficient mice (Ko et al., 2015).

Interestingly, Tet2 was first demonstrated to catalyze 5mC into 5hmC on mammalian RNA (Zhang et al., 2015). In the setting of pathogen infection, Tet2 can repress the mRNA expression of suppressor of cytokine signaling 3 (Socs3), a negative regulator of the Janus kinase (JAK)-STAT pathway, promoting the production of myeloid cells and proliferation of immune cells. Furthermore, Tet2 suppresses the mRNA level of Socs3 by mediating the binding of adenosine deaminase acting on RNA 1 (ADAR1) (Ko et al., 2010). This study shed light on the role of RNA modification in innate immunity and inflammation from an epitranscriptomic perspective (Zhang et al., 2015).

Notably, the functions of TET proteins in myeloid cells differ under diverse circumstances. For example, TET proteins can induce inflammation and promote a myeloid cell immune response instead of suppressing inflammation. Tet2, as a candidate binding partner of the zinc finger protein CXXC5, is recruited to cooperatively regulate the methylation of a 500–1000 base pair region within the CpG island of the Irf7 gene in plasmacytoid dendritic cells (pDCs). After viral infection, mice deficient in CXXC5 or – to a lesser extent – in Tet2 fail to show an early IFN response, and the related production of IFNα/β, CCL-5, tumor necrosis factor (TNF), and IL-12 is attenuated (Ma et al., 2017).

## Role of Tet2 in Autoinflammatory and Autoimmune Diseases

Autoinflammatory diseases and autoimmune diseases have attracted increasing attention. They share many similar characteristics; for example, the pathological processes in both target self-tissues and both are systemic diseases involved in chronic activation of the immune system in individuals with genetic susceptibility, eventually leading to tissue inflammation (Zen et al., 2013). Therefore, immune cells and the immune response, which can be regulated by Tet2, are involved in the development of autoinflammatory diseases and autoimmune diseases.

Recently, the direct role of Tet2- and Tet3-mediated epigenetic modification in B cell tolerance was uncovered. In addition to functions in DNA-demethylating activities, TET proteins can regulate transcriptional repression in immune cells by recruiting HDACs (Zhang et al., 2015; Xue et al., 2016). A series of studies have demonstrated that Tet2/Tet3 DKO in B cells is a key factor that contributes to the spontaneous hyperactivation of both B and T cells, autoantibody production and autoimmunity development (Tanaka et al., 2020). Additionally, given that CD86 overexpression in tolerant B cells caused them to break tolerance (Rathmell et al., 1998), collectively, Tet2 and Tet3 are essential for CD86 expression suppression in self-tolerant B cells and play important roles in autoimmunity prevention. Furthermore, Tet2/Tet3 DKO mice tend to develop systemic lupus erythematosus (SLE)-like autoimmunity disease; compared with typical SLE mouse models, Tet2/Tet3 DKO mice seem to have relatively mild disease (Li et al., 2017). Notably, Tet2/Tet3 DKO invariant natural killer T cells (iNKT cells) displayed developmental and proliferation dysregulation so that these cells showed an uncontrolled tendency toward the NKT17 lineage, along with increased expression of DNA methylation and impaired expression of T-bet and ThPOK genes encoding key lineage factors (Tsagaratou et al., 2017). Collectively, studies of the abnormal epigenetic profile observed in TET protein-regulated self-tolerance and autoimmunity can improve our understanding of autoinflammatory and autoimmune diseases.

Many autoinflammatory syndromes display the over production of mature proinflammatory cytokines, including IL-1β and IL-18. Subsequently, the inflammasome response and innate immune cells are activated (Alvarez-Errico et al., 2017). During the conditions of monogenic autoinflammatory diseases such as cryopyrin-associated periodic syndromes and familial Mediterranean fever, inflammasome-related genes are more prone to DNA demethylation than healthy control subjects in monocyte-to-macrophage differentiation and monocyte activation, and this demethylated progress can be mediated by Tet2 and NF-κB (Vento-Tormo et al., 2017).

Systemic lupus erythematosus (SLE) is a typical autoimmune disease affecting multiple organs and systems (Gatto et al., 2013; Zeng et al., 2017). The pathogenesis of SLE is related to the abnormal differentiation and activation of T cells induced by factors related to genetic susceptibility and epigenetic regulation, such as unbalanced differentiation of Th2 and Th17 cells (Kleinewietfeld et al., 2013), reduced function and quantity of Treg cells, and an abnormally increased proportion of Tfh cells (Wu et al., 2016). Additionally, dysregulation of other immune cells, such as DCs, B cells and macrophages, is also related to the development of SLE (Wu et al., 2017; Zeng et al., 2017). DNA demethylation mediated by Tet2, DNMT1, DNMT3A, and DNMT3B is essential for DC development and maturation (Zhang et al., 2014). Furthermore, IL-4, the key cytokine for DC differentiation, contributes to this differentiation process by regulating STAT6-mediated DNA methylation in a TET-dependent manner (Vento-Tormo et al., 2016).

Tet2 contributes to Th cell differentiation via DNA demethylation and regulation of cytokine gene expression in T cells. A study demonstrated that Tet2-induced DNA demethylation promotes high salt concentrations during Tfh cell differentiation and then functions in promoting SLE development. An *in vitro* study indicated that Tet2 plays a positive role in human Tfh cell differentiation by enhancing the expression of PD-1 and BCL-6. Among the genes activated by high salt concentrations and involved in T cell differentiation and activation, spn is directly regulated by Tet2, and this relationship may provide a novel treatment for SLE by controlling a high-salt diet and intervention with Tet2-mediated Tfh cell differentiation (Ichiyama et al., 2015; Wu et al., 2016; Yang et al., 2016). These observations suggest that a relationship exists between Tet2 and SLE (Wu et al., 2016).

Additionally, osteoclast activation is related to rheumatoid arthritis (RA); Tet2 can contribute to osteoclast differentiation and suppress inflammation, and a relationship between Tet2 and RA has been established (Scott et al., 2010). Additionally, the pathogenesis of multiple sclerosis (MS), a chronic autoimmune disease resulting in axonal degeneration and demyelination, is caused by the disruption of epigenetic profiles in immune system genes, accompanied by damage to the brain and spinal cord (SC). Peripheral blood mononuclear cells (PBMCs) from MS patients have an abnormal distribution of DNA methylation in the promoter region of specific genes (Calabrese et al., 2014). Patients with MS have significantly decreased expression of Tet1/2 and levels of 5hmC. Western blot and qPCR analysis showed that downregulated expression of both Tet1 and Tet2 primarily explains the decreased 5hmC level in experimental autoimmune encephalomyelitis (EAE)-induced injury. When EAE mice were treated with VC, the mRNA expression levels of Tet1 and Tet2 and the level of 5hmC increased, and the neurological behavioral deficits were reversed. Furthermore, Tet1 and Tet2 regulate the expression of BDNF (a key mediator of myelin repair and functional neurological recovery in MS) in EAE mice by enhancing 5hmC modification of the BDNF gene. Therefore, these results suggest that DNA methylation plays an important role in SC damage, although the detailed role of 5hmC modification in MS remains to be explored (Tang et al., 2019). Additionally, immunofluorescence analysis of the levels of Tet2, 5mC, 5hmC and DNMTs showed that, in Sjögren’s syndrome (SS) patients, the levels of both 5mC and DNMTs were decreased, while those of Tet2 and 5hmC were increasingly enriched in salivary gland inflammatory cells. Furthermore, cytokines activate DNA hydroxymethylation by inducing Tet2 enzyme expression, and this mechanism mediates the pathogenesis of SS (Lagos et al., 2018).

Type 1 diabetes (T1D) is an autoimmune disease directed against pancreatic islet β cells. Both genetic and environmental factors play key roles in the pathogenesis of T1D and eventually cause the loss of functional β cell mass and hyperglycemia (Xie et al., 2018). MiRNAs regulate TET expression posttranscriptionally, and miR-142-3p expression is induced in islet autoimmunity. Repression of miR-142-3p expression promotes the induction and enhances the stability of Treg cells. In mouse models of T1D, the miR-142-3p/Tet2/Foxp3 axis suppresses the induction of Treg cells and then impairs their stability. Mechanistically, Tet2 targets miR-142-3p directly, linking high miR-142-3p levels with epigenetic remodeling in Treg cells (Scherm et al., 2019). Additionally, in β cells of IFNα–INS1^*CreERT*2^ transgenic mice, IFN-α induces epigenetic modulation of DNA, also leading to T1D. Mechanistically, miR-26a targets the TET and TDG 3′-UTRs. After the degradation of miR-26a by PNPase old-35 (PNPT1), the posttranscriptional regulation of the Tet2 3’-UTR is altered accordingly, promoting Tet2 expression and 5hmC modification in β cells and resulting in DNA demethylation. Because of changes in the miR-26a/Tet2 regulatory network, the expression of Tet2 is upregulated by IFN-α (Stefan-Lifshitz et al., 2019). Additionally, the level of Tet2 is significantly increased in diabetic wounds, and Tet2 is associated with insulin sensitivity and the pathogenesis of diabetic nephropathy (Shen et al., 2018).

Pathogenic factors of atherosclerosis are also associated with other related autoimmune diseases (Lim et al., 2014). The function of Tet2 in atherosclerosis was also explored. Some reports have demonstrated that Tet2 acts as an important factor during the development of atherosclerosis. In a mouse model of low-density lipoprotein receptor deficiency, Tet2 loss of function preferentially resulted in the development of atherosclerosis (Fuster et al., 2017). A causal link between somatic Tet2 mutation-induced clonal hematopoiesis and exacerbated atherosclerosis has been suggested (Jaiswal et al., 2014). In mice, Tet2 participates in clonal hematopoiesis and leads to a large increase in atherosclerotic plaque size, which is correlated with the risk of atherosclerotic cardiovascular disease (CVD) (Ridker and Luscher, 2014).

Tet2 performs critical functions in regulating immune responses and DNA repair, maintaining genomic stability and enhancing tolerance or antitumor immunity. These results prove the key role of Tet2 in activating innate immunity and contribute to the identification of new effective therapeutic targets and approaches for infectious and inflammatory diseases (Shen et al., 2018).

## Harnessing the Power for the Light Side

In myeloid malignancies, the expression of the Tet2 gene and its variants was first identified in 2009. Subsequently, a series of studies focusing on the functions of Tet2 in hematologic malignancies were conducted. Because Tet2 is a common tumor suppressor gene, its lack of expression is always related to various human myeloid and lymphoid malignancies (Ko et al., 2010). The highest frequency of Tet2 mutation occurs in chronic myelomonocytic leukemia (CMML) (30–60%), followed by myelodysplastic syndrome (MDS) (20–35%) (Kosmider et al., 2009), acute myeloid leukemia (AML) (12–34%) (Grossmann et al., 2011), and lymphoid malignancies (2–33%) (Solary et al., 2014). Notably, no specific genotype patterns in the incidence of homozygous or heterozygous mutations are currently recognized in these diseases.

Recently, Tet2 has been hypothesized to enhance the sensitivity of myeloid leukemia cells to poly (ADP-ribose) polymerase inhibitors (PARPis) (Feng et al., 2019). Tet2 deficiency leads to impaired homologous recombination DNA repair and decreased expression of breast cancer susceptibility genes (BRCAs), and the reduction in BRCA gene expression sensitizes tumor cells to PARPis (Gojo et al., 2017; Feng et al., 2019). Therefore, this finding suggests that the pathogenic genotypes of Tet2 and BRCA mutations can be models for applying PARPis to leukemia treatment.

In the Mexican population with AML, the prevalence of Tet2 mutations is 11.8% and that of DNMT3A mutations is 2.7%, resulting in irregular DNA methylation patterns and changes in the transcriptional expression levels of 16 specific AML-associated genes. Among these genes, some may be tumor suppressors, and their silencing may induce the activation of oncogenes (Alvarez et al., 2013). Subsequently, a potential correlation between AML patient survival and activation of DNMT3A and Tet2 was reported, providing a novel strategy for improving AML patient survival by altering the normal methylation patterns of specific genes (Ponciano-Gomez et al., 2017). Notably, mutations in isocitrate dehydrogenase 1 and 2 (IDH1 and IDH2) also occur in 30% of AML patients. IDH and TET mutations are exclusive, although the hypermethylation signature of these genes overlaps in AML patients. In the setting of IDH mutation, 2-hydroxyglutarate (2-HG) may be produced; 2-HG can then compete with α-ketoglutarate to suppress α-OG-dependent Tet2 activity (Cimmino et al., 2017). Compared with patients with heterozygous Tet2 mutations, patients with homozygous Tet2 mutations show a decreased event-free survival (EFS) ratio and an increased recurrence rate (Ahn et al., 2015).

Additionally, that p53 regulates Tet2 stability through autophagic degradation pathways has recently been proposed. As a tumor suppressor, p53 is critical in maintaining DNA repair, cell cycle arrest and cell death programming in response to DNA damage (Bieging et al., 2014; Duffy et al., 2017). Tumor cells with p53 inactivation always show chemotherapeutic resistance. Mechanistically, P53 interacts with Tet2 and translocates it from the nucleus to the cytoplasm; Tet2 is then trapped in the cytosol and associates with autophagosomes before being degraded. These findings suggest that, in p53-null tumors, Tet2 inhibition is a potential strategy to restore drug sensitivity. Therefore, to fully exploit TET activators and agonists for cancer treatment, special attention should be devoted to this possibility (Zhang et al., 2019).

In MDS, Tet2 mutation not only causes DNA methylation but also results in the dysregulation of gene expression in hematopoietic stem/progenitor cells (HSPCs) as well as the specific proliferation of abnormal myeloid cells (Lin et al., 2014). In MDS HSPCs, the protein level of sirtuin 1 (SIRT1) is decreased. The relationship between SIRT1 and Tet2 in MDS HSPCs was discovered based on the observation that SIRT1 plays an important role in stem cell proliferation, survival and self-renewal (Chalkiadaki and Guarente, 2015). Mechanistically, SIRT1 deacetylates Tet2 at conserved lysine residues in its catalytic domain, resulting in enhanced Tet2 activity. Moreover, activated SIRT1 functions as an inhibitor of Tet2-mediated MDS cell growth, while the SIRT1/Tet2 axis regulates a series of cancer-related genes. Collectively, these findings indicate that SIRT1 activation can be considered a novel therapeutic approach to regulate the activity of Tet2 in MDS HSPCs and support more detailed explorations of the SIRT1/Tet2 axis as a potential opportunity for exploitation in MDS (Sun et al., 2018).

Chimeric antigen receptor T (CAR-T) cells have recently been considered an efficient therapeutic agent for B lymphocyte malignancies. During treatment, T cells are isolated from patients and transformed *in vitro* (by means such as chronic virus infection) to express CARs. When the modified cells are transferred back into the body, the CARs can induce T cells to specifically target tumor cells. Thus, this therapy is very effective for CD19-expressing B-lymphocytic leukemia and DLBCL (Fraietta et al., 2018). Studies have shown that Tet2 elimination can change the epigenetic map of cells and promote the proliferation of CAR-T cells derived from a single cell clone, thereby promoting remission in leukemia patients and improving the efficacy of immunotherapy (Fraietta et al., 2018).

## Conclusion and Perspectives

Ten-eleven translocation proteins, particularly Tet2, encode DNA demethylases that play indispensable roles in stem cell differentiation and reprogramming to pluripotency in every developmental stage of the immune system component discussed above. Tet2 regulates the expression of genes that determine cell fate. These genes encode key transcription factors such as T-bet and cytokines such as Ifng and Il4/Il17 in T cells; IRF4, E2A and PU.1 in B cells; and IL-6 and Socs3 in myeloid cells. By influencing the expression of these factors, Tet2 loss has indirect effects on the regulatory networks in which these factors participate, resulting in amplification of effects and further deregulation of cell type-specific gene expression programs (Table 1). Although numerous studies have shown the important functions of Tet2 in gene regulation, cell differentiation and tumor suppression, the detailed mechanisms of Tet2 must be further studied and clarified, such as the following: ➀ a specific mechanism of the Tet2- and TDG-regulated selective recruitment of histone-modifying enzymes to target specific genomic regions; ➁ the degree to which enzymatic activity-dependent and enzymatic activity-independent (structural) mechanisms contribute to Tet2 functions; ➂ the detailed mechanism of Tet2 in promoting malignancy and the relationship of Tet2 mutation with tumor prognosis; ➃ whether 5hmC and other oxidized methylcytosines perform other functions in epigenetic marking in addition to being intermediate products of DNA demethylation; ➄ the different mechanisms of Tet2 and DNMT3A mutations in hematopoiesis. In summary, to beneficially exploit Tet2 to its fullest role, it is necessary to understand the definite molecular mechanisms and specific targets of Tet2 and strategies to alter and achieve the desired magnitude and direction of immune responses and further regulate the related diseases.

**TABLE 1:** Summary of the currently identified Tet2-interacting molecules.

Interactors or regulators	Cell resource	Functions or alterations	References
**C/EBPα, Klf4 and Tfcp2l1**	Pluripotent stem cells	Interact with Tet2 to promote enhancer demethylation	Sardina et al., 2018
**IDAX (CXXC4)**	Human cell line and mouse ESCs	Binds Tet2 and DNA, decreases the protein level of Tet2	Tahiliani et al., 2009
**E2A and PU.1**	Pro-B cells	Recruit Tet2 to Eκ enhancers and maintain the demethylated status of these enhancers	Ghisletti et al., 2010
**IRF4**	B cells in the pro-B to pre-B transition	Is regulated by Tet2, restores Igκ transcription and demethylation	Lio et al., 2016
**EBFI**	Human B cells	Binds Tet2 to regulate DNA demethylation	Guilhamon et al., 2013
**AID**	Murine B cells	Is upregulated by Tet2, participates in somatic hypermutation and Ig class switching, affects the cellular distribution of TET enzymes	Pfaffeneder et al., 2014
**T-bet**	Th1 cells	Coordinates with Tet2 to regulate ifng expression	Ichiyama et al., 2015
**Il17 and Il17f**	Th17 cells	Tet2 induces DNA demethylation at the Il17-17f locus in a RORγt-dependent manner	Wang et al., 2012
**Il10**	Th17 cells	Tet2 is recruited to the CNS (2) at the Il10 locus in a STAT3-dependent manner	Wang et al., 2012
**Eomes and T-bet**	CD8^+^ T cells	Are regulated by Tet2, promote CD8^+^ T cell memory transcriptional programs	Carty et al., 2018
**H2S**	Foxp3^+^ Treg cells	Sulfhydrates NFYB to enhance its binding to the Tet1 and Tet2 promoters, H2S deficiency results in downregulation of Tet1 and Tet2	Li et al., 2014; Yang et al., 2015
**Smad3 and Stat5**	Foxp3^+^ Treg cells	Recruit Tet1 and Tet2 to bind to Foxp3 when activated	Yang et al., 2015
**Vitamin C**	TGF-β-induced Treg cells	Promotes folding of Tet2 enzymes and/or recycling of the cofactor Fe2^+^, increases the stability of Foxp3 expression in TGF-β-induced Treg cells	Ko et al., 2013; Someya et al., 2017
**TDG**	Bone marrow myeloid cells	Its downregulation in combination with Tet2 downregulation impairs the DNA methylation and recruitment of differential histone-modifying enzymes	Garcia-Gomez et al., 2017
**OGT**	Human cell line and mouse ESCs	Is recruited to chromatin by Tet2, promotes the activity of the H3K4 methyltransferase SETD1A and gene transcription	Bachman et al., 2014
**HDAC2**	myeloid cells	Tet2 recruits HDAC2 to specifically repress IL-6 for resolving inflammation	Zhang et al., 2015
**CD86**	self-tolerant B cells	Is suppressed by Tet2 and Tet3 and then plays roles in autoimmunity prevention	Tanaka et al., 2020
**Tet2, Tet3**	iNKT cells	TET proteins regulate the lineage specification and TCR-mediated expansion of iNKT cells	Tsagaratou et al., 2017
**miR142-3p**	Treg cells	Is directly targeted by miR142-3p, the miR142-3p/Tet2/Foxp3 axis inhibits the induction and decreases the stability of Treg cells	Scherm et al., 2019
**miR-26a**	β cells from IFNα–INS1^*CreERT2*^ transgenic mice	Targets the TET and TDG 3′-UTRs, PNPT1/miR-26a/Tet2 triggers autoimmune diabetes	Stefan-Lifshitz et al., 2019
**SIRT1**	HSPCs	Deacetylates Tet2 at conserved lysine residues in its catalytic domain and modulates Tet2 activity	Sun et al., 2018

*IDAX, inhibition of the Dvl and Axin complex; CXXC4, CXXC finger protein 4; AID, activation-induced cytidine deaminase; H2S, hydrogen sulfide; TDG, thymine-DNA glycosylase; OGT, O-GlcNAc transferase; PNPT1, PNPase old-35; TGF-β, transforming growth factor-β; Eomes, eomesodermin.*

## Author Contributions

JL and LL performed the literature search, wrote the first draft of the manuscript, and revised the text. XS, TD, GH, XL, and ZZ critically revised the text and provided substantial scientific contribution. ZX proposed the project and revised the manuscript. All authors approved the final version of the manuscript.

## Conflict of Interest

The authors declare that the research was conducted in the absence of any commercial or financial relationships that could be construed as a potential conflict of interest.
